# Difficult removal of exposed peripheral nerve stimulator leads: a report of 2 cases

**DOI:** 10.1097/PR9.0000000000000946

**Published:** 2021-08-09

**Authors:** Pushpinder Uppal, Thelma B. Wright, Layth Dahbour, Blake Watterworth, Seung J. Lee, Kanchana Gattu, Lynn G. Stansbury, Justin Benoit

**Affiliations:** a Department of Anesthesiology, University of Maryland Medical Center, Baltimore, MD, USA; b Shock Trauma Associates, University of Maryland Medical Center, Baltimore, MD, USA

**Keywords:** Case report, Stimulator lead migration, Peripheral nerve stimulator, PNS, Complications

## Abstract

**Introduction:**

Peripheral nerve stimulators have emerged as a new generation of advanced modalities to treat chronic pain and avoid opioids. They transmit electrical stimulation through implanted leads and wireless, wearable, external generators. Common complications include infection, nerve damage, and migration of stimulating leads. This article describes 2 cases of complications from lead migration.

**Methods:**

Case 1 describes a 61-year-old man with chronic groin pain who underwent an uncomplicated ultrasound-guided ilioinguinal peripheral nerve lead implantation. Case 2 describes a 54-year-old woman with left shoulder pain who underwent an uncomplicated ultrasound-guided percutaneous lead placement near the axillary nerve through a deltoid approach. Both peripheral nerve stimulators were confirmed with fluoroscopy, and each patient was followed up every 2 months for the following 2 years.

**Results:**

Both patients experienced lead migration to the skin resulting in erythema and need for lead removal. Initial unsuccessful removal by traction resulted in retained fragments and need for open surgical removal.

**Discussion:**

Neurologic complications of peripheral nerve stimulator implantation are rare, but device-associated complications, specifically lead migration, remain a source of long-term problems that can result in decreased coverage of the intended neural target.

**Conclusion:**

Thorough patient education, early postimplantation assessment, and extended routine follow-up are necessary to decrease lead-associated complications. If migration does occur, the potential impact of scar tissue on removal should be considered.

## Abstract 2

Peripheral nerve stimulators serve as an alternative modality to treat chronic pain conditions; however, long-term complications, specifically lead migration, may occur.

## 1. Introduction

The demand for innovative technologies to treat chronic pain effectively and safely has fostered a new generation of peripheral nerve stimulation devices. Historically, these devices have relied on insertion of an implantable pulse generator as well as the peripheral nerve stimulation lead.^1^ These generators can be relatively bulky and require intermittent revision to replace batteries. Newer systems now incorporate technology that transmits electrical stimulation through wearable, wireless, external generators.^5^ This reduces the invasiveness of the procedure and avoids the periodic need for surgical removal of the generator, overall decreasing the likelihood of surgical complications.^3^ With the current demand for strategies to decrease opioid use in the treatment of chronic pain, interest in these electrical neuromodulation techniques has increased.^7^ Stimulators are being adopted to treat many conditions, from chronic regional pain syndrome to occipital headaches.^4^ However, as more of these devices are implanted, the incidence and range of complications will inevitably rise.

We present 2 cases of peripheral nerve stimulator lead complications. In both cases, the leads migrated, became partially exposed, failed the remediation procedure advised by the manufacturer, and required surgical removal. The patients provided written HIPAA-compliant permission for the inclusion of their clinical information in this report.

### 1.1. Case 1—methods

A 61-year-old man presented with chronic left groin pain after left inguinal hernia repair several years earlier. He described this pain as 9 of 10 per the Numerical Rating Scale in intensity, sharp, and shooting, with radiation to the abdomen. Pharmacological therapies, including anticonvulsants, NSAIDs, and opiates, had proven inadequate. An ilioinguinal peripheral nerve block was performed for diagnostic and prognostic purposes. This procedure reduced reported pain intensity to 1 on the Numerical Rating Scale, and the patient elected to proceed with implantation of an ilioinguinal peripheral nerve stimulator for ongoing pain control.

Lead implantation was performed at an outpatient surgery center using monitored anesthetic care and standard sterile surgical technique. Ultrasound guidance was used to visualize the ilioinguinal nerve between the oblique and transversus abdominus muscles. A percutaneous StimRouter (Bioness, Inc., CA) peripheral nerve stimulation system was used for the procedure. Intraoperative mapping of pain coverage was confirmed. The device was anchored per manufacturer recommendations, and fluoroscopy was used to confirm final placement. In follow-up, the patient reported adequate pain control, with pain intensity decreasing by 50% using the device.

### 1.2. Case 1—results

**Figure 1.**
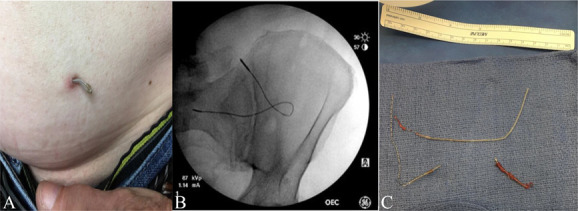
Case 1. (A) Peripheral lead from ilioinguinal nerve stimulator protruding from the skin. (B) X-ray of a peripheral stimulator before removal in OR. (C) Peripheral stimulator lead which fractured during removal.

The patient was evaluated every 2 months after the procedure. At his 12-month follow-up, the patient revealed a loop of exposed electrode protruding from his lower abdomen (Fig. 1A). He was uncertain when the lead had become exposed in the interval since his last follow-up. The decision was made to explant the device because of erythema at the site and loss of adequate analgesic benefit. The manufacturer was informed of the complication and provided recommendations for removal technique.

Accordingly, initial attempts were made to remove the lead by traction; however, this was unsuccessful (Fig. 1B). The lead seems to be tightly anchored to the underlying fascia by incorporated fibrous tissue. We progressed to an open procedure. This ultimately required a 2-inch incision and dissection of the muscle layer for complete extraction of the device. During removal, the lead was noted to have also fractured (Fig. 1C). Fluoroscopy was used to confirm that no fragments of the lead were retained. The area was generously irrigated and closed in standard fashion. The patient had no complications postprocedure.

### 1.3. Case 2—methods

A 54-year-old woman presented with left shoulder pain after an ischemic stroke 5 years before. Given the persistence and intensity of her pain, the patient underwent an uncomplicated permanent placement of the percutaneous StimRouter (Bioness, Inc.) device targeting the axillary nerve. Ultrasound guidance was used to direct lead placement. Intraoperative mapping of pain coverage was confirmed. The device was anchored per manufacturer recommendations, and fluoroscopy was used to confirm final placement. A single 3-0 Vicryl suture was used to approximate the puncture incision, and skin adhesive was applied (Dermabond; Ethicon, Somerville, NJ) to the puncture and distal tunneling sites. These sites were then covered with 4 × 4s and a Tegaderm. Her recovery was uneventful, and she reported 50% pain relief with the device.

### 1.4. Case 2—results

**Figure 2.**
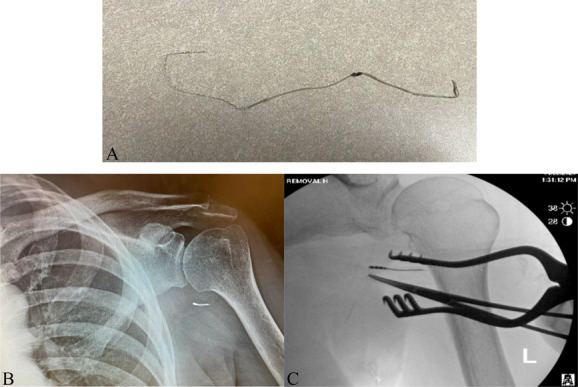
Case 2. (A) Peripheral stimulator lead removed in clinic. (B) X-ray with retained PNS lead fragment. (C) Medial migration of PNS lead fragment during attempted surgical removal in the operating room.

Three years after implantation, the patient returned presenting a lead protruding through the skin at the lead insertion site and loss of analgesia. We attempted device removal using simple traction, which seemed to be successful (Fig. 2A). Radiographic evaluation, however, revealed retention of a lead fragment (Fig. 2B).

The following day, the patient was taken to the operating room for open removal of the retained fragment. The fragment had migrated deep to the original implantation site, approaching neurovascular structures of the brachial plexus (Fig. 2C). Given the risk of injury to surrounding structures, the procedure was aborted, and the incision was closed. The patient tolerated the procedure well and has had an uneventful recovery.

## 2. Discussion

Neuromodulation has emerged as a key pain management tool, and the technology of peripheral nerve stimulation has advanced significantly over the past 20 years. Percutaneous implantation techniques with smaller devices, rechargeable batteries, and larger capacity batteries have all contributed to this advance.^5^ Older spinal cord stimulator devices incurred frequent adverse events, mainly associated with implantation. A 2004 review summarizing 68 reports of the previous 20 years' experience with these devices noted 16% overall adverse events, including hardware erosion (7%), hardware migration (2%), hardware failure (2%), lead infection (1%), and other lead-related events (4%).^1^ A major presumption of the newer generation of peripheral nerve stimulators has been that, without the need for implanted generator hardware, the incidence of implantation-associated events would decrease.

Deer et al.^2^ described the safety and efficacy of one such peripheral nerve stimulation device. Using a crossover design, 94 patients met the inclusion criteria to undergo nerve stimulator lead implantation. Forty-five patients underwent the 3-month primary trial, whereas the control subjects continued with standard medical treatments. Differences in adverse events between groups were reported as nonsignificant. These adverse events did not seem to be related to delayed effects of lead placement. Of note, follow-up was limited to 12 months, and more than half (48/94, 51%) of enrolled patients were lost to follow-up after 6 months.

By contrast, several recent reviews suggest that these devices can have long-term complications not identified within standard trial intervals.^3,6^ Eldabe et al. note that although neurologic complications are rare, device-associated complications remain an important source of long-term problems, the most common being migration of the stimulator lead. This in turn diminishes the treatment response at the intended neural target^3^ as was true for the patient in Case 1, after the lead protruded through his skin. Verrills et al. state that complications of spinal cord and peripheral nerve stimulators have been reported by 30-40% of patients.^6^ Skin erosion was one commonly reported issue, which was the experience in both Case 1 and Case 2.

Surgical and clinical success with peripheral nerve stimulation starts with patient selection. Patients must be free of major psychiatric disease to be able to participate fully in diagnostic and therapeutic procedures and in follow-up plans. Pain distribution reported by patients and confirmed by physical examination must be consistent with the sensory distribution of a single peripheral nerve. Entrapment neuropathies should be excluded. A positive response to diagnostic peripheral nerve block^5^ may also support the decision to use peripheral stimulation.

After permanent implantation, patients should be provided specific postoperative education to avoid activities that may contribute to lead migration. Sufficient local scarring usually occurs in 3 months and is helpful to reinforce anchoring. Research and clinical experience suggest that education regarding remote lead migration, signs of localized skin breakdown, and the need for long-term follow-up should be incorporated into routine care plans.^3^

## 3. Conclusion

Peripheral nerve stimulation can be a useful modality for the treatment of chronic pain of peripheral nerve origin. As with all implantable devices, the treating physician and patient should be aware of long-term complications associated with use. We describe 2 cases of lead migration that resulted in system failure. These can be remote complications to the procedure, so long-term monitoring of devices is essential. If complications do arise secondary to lead migration, factors that may lead to difficult lead extraction must be considered, and the position and integrity of the system must be fully evaluated.

## Disclosures

The authors have no conflicts of interest to declare.
